# Dietary supplementation of mannan-oligosaccharide enhances neonatal immune responses in chickens during natural exposure to *Eimeria *spp

**DOI:** 10.1186/1751-0147-51-11

**Published:** 2009-03-19

**Authors:** Gabriela Gómez-Verduzco, Arturo Cortes-Cuevas, Carlos López-Coello, Ernesto Ávila-González, Gerardo M Nava

**Affiliations:** 1 Facultad de Medicina Veterinaria y Zootecnia, Universidad Nacional Autónoma de México, 04510, México City, México; 2 Current Address: University of Illinois at Urbana-Champaign, USA, 1207 W Gregory, Urbana, IL 61801, USA

## Abstract

**Background:**

Control and eradication of intestinal infections caused by protozoa are important biomedical challenges worldwide. Prophylactic control of coccidiosis has been achieved with the use of anticoccidial drugs; however, the increase in anticoccidial resistance has raised concerns about the need for new alternatives for the control of coccidial infections. In fact, new strategies are needed to induce potent protective immune responses in neonatal individuals.

**Methods:**

The effects of a dietary supplementation of mannan-oligosaccharide (yeast cell wall; YCW) on the local, humoral and cell-mediated immune responses, and intestinal replication of coccidia were evaluated in a neonatal animal model during natural exposure to *Eimeria *spp. A total of 840 one-day-old chicks were distributed among four dietary regimens: A) Control diet (no YCW) plus anticoccidial vaccine); B) Control diet plus coccidiostat; C) YCW diet plus anticoccidial vaccination; and D) YCW diet plus coccidiostat. Weight gain, feed consumption and immunological parameters were examined within the first seven weeks of life.

**Results:**

Dietary supplementation of 0.05% of YCW increased local mucosal IgA secretions, humoral and cell-mediated immune responses, and reduced parasite excretion in feces.

**Conclusion:**

Dietary supplementation of yeast cell wall in neonatal animals can enhance the immune response against coccidial infections. The present study reveals the potential of YCW as adjuvant for modulating mucosal immune responses.

## Background

Control and eradication of intestinal infections caused by protozoa are important biotechnological and medical challenges worldwide. The coccidial parasites *Eimeria*, *Toxoplasma*, *Neospora *and *Cryptosporidium *are the cause of serious diseases in humans and animals. Many of these intracellular protozoa affect the small intestine and produce devastating effects on immune compromised subjects [1-3]. These parasitic infections can cause a wide range of harm to the infected host; from diarrhea to abortion, death or chronic nutritional and cognitive sequels [4,5]. Coccidial infections have been controlled, to a great extent, with the use of anticoccidial drugs; however, the increase in resistance to many of these products has raised concerns about the need for new anticoccidial alternatives [4]. A challenging factor is that protozoan parasites multiply inside of host cells; thus, cell mediated immune responses are required for protective immunity [4,5]. Therefore, development of effective anticoccidial strategies requires substantial knowledge of the parasite biology and host-parasite interactions [4].

Progress on new anticoccidial strategies has been accomplished in livestock and poultry. In these animal species, protozoan intestinal infections are an important factor associated with poor performance and production efficiency [4,6,7]. Avian coccidiosis, caused by the apicomplexan protozoan *Eimeria*, is the major parasitic disease of poultry. For the world poultry industry, the cost of coccidiosis excess USD 3 billion annually [6].

Currently, prophylactic protection against coccidiosis in humans and animals can be accomplished with the use of anticoccidial vaccines. However, the use of live vaccines has been gradually adopted mainly due to economic reasons such as elevated cost of vaccination and adverse effects on early growth [4,8-10]. Even though research and development of effective vaccine formulations are still in progress [11,12], there is a need for new studies focused on the design of novel strategies to induce potent protective immune responses without adverse effects in neonate or immune-compromised individuals [4,13,14]. Here, a neonatal animal model was used to examine the effect of a dietary supplementation of mannan-oligosaccharide (in the form of yeast cell wall (YCW) on the local and humoral immune responses in neonatal chickens during natural exposure to coccidia parasite. This study revealed that dietary supplementation of 0.05% of YCW increases local, cellular and humoral immune responses, and reduces parasite excretion in feces.

## Methods

### Experimental design and animals

A total of 840 one-day-old Ross 308 chicks were distributed randomly in 28 pens bedded with fresh wood shavings (30 chickens per pen; 1:1 female/male ratio) in an experimental poultry house. The temperature was 32°C in each room on day 1 and was decreased weekly until the minimum temperature of 21°C was reached. Chickens were fed conventional starter (1 to 21 days old) and finisher (22 to 49 days) sorghum/soy based diets that met National Research Council requirements (Nutrient requirement of poultry, 1994). Fluorescent lighting was provided under the following schedule: day 0–2: 23 h light and 1 h dark, day 3–11: 18 h light and 6 h dark; day 12-end: 16 h light and 8 h dark. Chicks were allowed to consume feed and water *ad libitum *during the entire time of the experiment. The experiments were performed under poultry farm conditions, where animals are naturally exposed to different species of *Eimeria *(i.e. *E. acervulina, E. maxima *and *E. tenella*; [13]). Experimental procedures were approved by the Institutional Committee for Care and Use of Experimental Animals at the Facultad de Medicina Veterinaria y Zootecnia, Universidad Nacional Autónoma de México.

Basal diets were formulated without antibiotics and were identical across treatments with the following exceptions: treatments B and D included coccidiostat [125 ppm of nicarbazin (Helm, Naucalpan, Mexico) for starter diet and 65 ppm of salinomycin (Helm, Naucalpan, Mexico) for finisher diet]. Treatments C and D included 0.05% (w/w) of YCW (a concentrate of yeast hulls obtained via autolysis of *Saccharomyces cerevisiae *(Safmex, Toluca, Mexico). At arrival, chicks were randomly divided among treatments. The experimental design was a factorial arrangement of treatments, consisting of four dietary regimens: A) Control diet (no YCW) plus anticoccidial vaccine); B) Control diet (no YCW) plus coccidiostat; C) YCW diet plus anticoccidial vaccination; and D) YCW diet plus coccidiostat [7 replicates (pens) of each group, for a total of 210 animals per treatment]. Animals from the vaccinated groups were individually vaccinated at day 1 of age with a single oral dose of a commercial vaccine against coccidia (Advent cocci-vaccine; Viridus Animal Health LLC – Novus International Inc., St. Louis, MO) following the manufacturer's specifications. Animal weight gain and feed consumption were recorded at 21 and 49 days of age. At the end of the experimental period (49 days), 20 birds per treatment were euthanized by cervical dislocation. Carcass yield and breast skin pigmentation (yellow pigmentation after cold processing analyzed by reflectance colorimetric, Minolta-CR 400) were measured.

### Assessment of the humoral immune response

The effect of different treatments on humoral immune response was examined by means of antibody production after a simultaneous vaccination against Newcastle disease virus (NDV). Ocular (eye drop 0.03 ml active virus, Nobilis ND Lasota, Intervet/Schering-Plough Animal Health; Mexico) and subcutaneous (0.25 ml inactivated virus, Intervet/Schering-Plough Animal Health) vaccines were administered at 10 days of age. Serum samples were taken at 7 and 14 days post-vaccination (35 samples per treatment), frozen at -20°C, and specific antibody titers against NDV were determined by hemagglutination inhibition assay [15].

### Assessment of the mucosal immune response

The effect of different treatments on mucosal immune response was examined by means of production of secretory immunoglobulin A (IgA). Duodenum and tracheal samples (10 chickens per treatment) were collected at 21 days of age to measure production of secretory IgA. Ten centimeters of duodenum were taken and mucosal lavages were performed with 10 ml of cold and sterile phosphate-buffered saline solution (PBS). Tracheal samples were collected by tracheal swabs and placed (12 hrs) in tubes containing 10 ml of sterile PBS. All mucosal samples were clarified by centrifugation (1200 × g) and frozen at -20°C. Secretory IgA concentrations were measured by enzyme-linked immunosorbent assay (ELISA) test (chicken IgA ELISA Quantitation Kit, Bethyl Laboratories Inc. Montgomery, TX) following the manufacturer's protocol [16].

### Assessment of the cell-mediated immune response

The effect of different treatments on cell-mediated immune response was examined by cutaneous basophilic hypersensitivity test [17,18]. Analysis of cell-mediated immune response at 21 days was performed as descried elsewhere [17]. Briefly, an intradermic inoculation of phytohemagglutinin (PHA-A3; 0.1 mg/0.1 ml) was carried out in the inter-digital membrane of 3^rd ^and 4^rd ^phalanges of the right inferior extremity and sterile saline solution (0.1 ml) of the left foot (35 chickens per treatment). Twenty-four hours post-inoculation the thickness of the inter-digital membrane was measured in millimeters using a micrometer.

### Assessment of the replication of coccidia in the intestinal tract

The effect of different treatments on the replication of coccidia in the intestinal tract was estimated by quantifying the number of oocysts excreted in feces. At 21 days of age, ten birds per treatment were placed in separate cages with clean sheets of paper on the bottom. Fecal samples were collected from the paper lining after an 8-hour period. Birds were returned to their respective pens at the end of the fecal collection period. The total number of oocysts was calculated following the method described previously [19].

### Statistical analysis

Results were statistically analyzed by ANOVA in a complete randomized design with 2 × 2 factorial arrangement and by the GLM procedure of the SAS software (SAS Institute, Cary, NC). Data of animal performance and immunological parameters were grouped and presented as mean ± SEM. *P*-values ≤ 0.05 were considered statistically significant. Two factors were examined and compared across the study, 1) diet: control sorghum/soy-based diet (Control) vs. YCW diet (YCW); and 2) anticoccidial program: anticoccidial vaccine (Vaccine) vs. coccidiostat drug (Coccidiostat).

## Results

### Animal growth

The effects of diet and anticoccidial program on animal performance at 21 and 49 days of age are described in Table 1 and 2, respectively. At 21 days of age (Table 1), YCW increased weight gain and feed consumption compared to Control (*P *< 0.01). However, no significant differences in feed conversion were observed between Control and YCW (*P *> 0.05). Also, it was found that Coccidiostat increased weight gain and feed consumption compared to Vaccine (*P *< 0.01), and that Vaccine increased feed conversion (*P *< 0.05). These results reveal the common adverse effects on growth of chicks caused by anticoccidial vaccination [8]. In the analysis at 49 days of age (Table 2), YCW increased weight gain and reduced feed conversion compared to Control (*P *< 0.05). There were no differences in percentage of carcass yield and skin pigmentation between Control and YCW (*P *> 0.05; data not shown). Also, there were no differences (*P *> 0.05) in weight gain, feed consumption, feed conversion (Table 2), percentage of carcass yield and skin pigmentation between Coccidiostat and Vaccine factors at day 49 of age (data not shown).

**Table 1:** Effect of yeast-cell-wall (YCW) diet on growth in chickens during the first 3-weeks of age under coccidial infection

	**Weight gain (g)**	**Feed consumption (g)**	**Feed conversion**
**Diet**			
**Control**	750 ± 14^b^	1009 ± 10.27^b^	1.34 ± 0.03
**YCW**	779 ± 16^a^	1047 ± 16.91^a^	1.34 ± 0.02
**Anticoccidial program**			
**Coccidiostat**	805 ± 14.0^a^	1051 ± 12.47^a^	1.30 ± 0.02^b^
**Vaccine**	725 ± 8.4^b^	1005 ± 20.13^b^	1.38 ± 0.03^a^
**Variation source**	**Probability**		
Diet	0.001	0.007	0.965
Anticoccidial program	0.037	0.019	0.005
Sample size per group	210	210	210

Two factors were examined and compared across the study, 1) diet: control diet (Control) vs. YCW diet (YCW); and 2) anticoccidial program: anticoccidial vaccine (Vaccine) vs. coccidiostat drug (Coccidiostat). Values in the same column with different superscripts (within factors) are statistically significant. Mean + SEM. *P*-values ≤ 0.05 were considered statistically significant.

**Table 2:** Effect of yeast-cell-wall (YCW) diet on growth in chickens during the first 7-weeks of age under coccidial infection

	**Weight gain (g)**	**Feed consumption (g)**	**Feed conversion**
**Diet**			
**Control**	3060 ± 20.98^b^	5883 ± 58.67	1.92 ± 0.02^a^
**YCW**	3135 ± 38.21^a^	5854 ± 44.39	1.85 ± 0.04^b^
**Anticoccidial program**			
**Coccidiostat**	3125 ± 39.42	5902 ± 51.41	1.87 ± 0.02
**Vaccine**	3069 ± 43.40	5835 ± 60.85	1.90 ± 0.03
**Variation source**	**Probability**		
**Diet**	0.045	0.227	0.021
**Anticoccidial program**	0.125	0.614	0.656
**Sample size per group**	210	210	210

For details see legend in Table 1. Values in the same column with different superscripts (within factors) are statistically significant. Mean + SEM. *P*-values < 0.05 were considered statistically significant.

### Assessment of humoral immune response

The effect of different treatments on the humoral immune response was examined by means of antibody production after a simultaneous vaccination against NDV. Ocular and subcutaneous vaccines were administered at 10 days of age and antibody production was measured at 7 and 14 days post-vaccination (Table 3). At 14 days of age, YCW induced higher NDV antibody titers compared to Control (*P *< 0.01). There were no differences in antibody titers between Vaccine and Coccidiostat (*P *> 0.05). The immune-regulatory effect was also observed at 21 days of age. YCW induced the higher NDV antibody titers compared to Control (*P *< 0.01). No significant effects were observed between Vaccine and Coccidiostat factors (*P *> 0.05). These results reveal that YCW enhances the humoral immune response against vaccine antigens in young animals.

**Table 3:** Effect of yeast-cell-wall (YCW) diet on IgA production in mucosa and humoral immune responses to Newcastle disease virus (NDV) in neonatal chickens

	**Intestinal IgA (ng/ml)**	**Tracheal IgA (ng/ml)**	Antibody titers* against NDV **17 days**	**Antibody titers against NDV** **24 days**
**Diet**				
**Control**	622 ± 99.70^b^	83.61 ± 12.5^b^	3.11 ± 0.16^b^	5.55 ± 0.08^b^
**YCW**	1281 ± 152.1^a^	169.70 ± 13.2^a^	3.86 ± 0.05^a^	7.71 ± 0.09^a^
**Anticoccidial program**				
**Coccidiostat**	450 ± 55.40^b^	145.55 ± 33.8	4.28 ± 0.58	6.57 ± 0.12
**Vaccine**	585 ± 143.2^a^	107.76 ± 20.2	4.07 ± 0.17	7.15 ± 0.09
**Variation source**	**Probability**			
**Diet**	0.009	0.011	0.015	0.005
**Anticoccidial program**	0.006	0.224	0.187	0.209
**Sample size per group**	10	10	35	35

For details see legend in Table 1. Values in the same column with different superscripts (within factors) are statistically significant. Mean + SEM. *P*-values < 0.05 were considered statistically significant. *Log2.

### Assessment of mucosal immune response

The effect of different treatments on mucosal immune response was examined by means of production of secretory IgA. Results of the IgA concentrations are presented in Table 3. In duodenal samples, YCW and Vaccine increased mucosal concentrations of intestinal IgA compared to Control and Coccidiostat, respectively (*P *< 0.01). In tracheal samples, YCW increased concentrations of mucosal IgA compared to Control (*P *< 0.05). No significant differences were observed between Vaccine and Coccidiostat.

### Assessment of cell-mediated immune response

The effect of different treatments on cell-mediated immune response was examined by the cutaneous basophilic hypersensitivity test. This method reveals the status of T-cell response. Results of the cell-mediated immune response at 21 days are presented in Table 4. YCW increased cell-mediated immune response compared to Control (*P *< 0.05). No significant differences for this parameter were observed between Vaccine and Coccidiostat (*P *> 0.05). These results confirm the systemic immune-regulatory effects of YCW and reveal that this compound enhances cell-mediated immune responses in young animals.

**Table 4:** Effect of yeast-cell-wall (YCW) diet on cell-mediated immune responses and fecal excretion of coccidia oocysts in neonatal chickens

	**Basophilic hypersensitivity (mm^2^)**	**Oocyst in feces (cells/g)**
**Diet**		
**Control**	0.56 ± 0.08^a^	32805 ± 572.80^a^
**YCW**	0.82 ± 0.09^b^	6505 ± 128.06^b^
**Anticoccidial program**		
**Coccidiostat**	0.67 ± 0.12	2810 ± 2454.36^b^
**Vaccine**	0.71 ± 0.09	36500 ± 2308.66^a^
**Variation source**	**Probability**	
**Diet**	0.012	0.001
**Anticoccidial program**	0.682	0.001
**Sample size per group**	35	10

For details see legend in Table 1. Values in the same column with different superscripts (within factors) are statistically significant. Mean + SEM. *P*-values < 0.05 were considered statistically significant.

### Assessment of the replication of coccidia in the intestinal tract

The effect of the different treatments on the replication of coccidia in the intestinal tract was verified by quantifying the number of oocysts excreted in feces. Results of the oocyst count at 21 days of age are presented in Table 4. YCW reduced the number of oocysts excreted in feces compared to Control (*P *< 0.01). Also, as expected, Coccidiostat reduced oocyst excretion compared to Vaccine (*P *< 0.05). These results reveal that the immune-regulatory effects produced by YCW reduce the replication of coccidia in the intestinal tract of young animals.

## Discussion

The development of new strategies for control of coccidiosis is essential for the poultry industry. Chickens are highly at risk for coccidial infections due to environmental conditions during production. High animal densities (> 25,000 chickens per building) on floor pens and warm surroundings are favorable for a high transmission, replication and accumulation of *Eimeria *spp. [13]. Moreover, the current practices for animal production create a strong selective pressure on coccidia parasites to develop anticoccidial drug resistance [20]. Together, these factors make neonatal chicken an excellent animal model for the study and design of new and effective strategies for the control of coccidiosis.

A current strategy for the control of avian coccidiosis is the use of live anticoccidial vaccines; however, this approach has been gradually accepted because of high cost of the vaccine and some adverse effects on early animal growth observed after vaccination [8]. In the present study, the vaccinated group showed a negative effect on chicken growth rates during the first 3-weeks of age; nonetheless, by 7-weeks of age, this group had a compensatory growth and the animal performance was similar among all groups by the end of the experiment. In addition, the data of the present study revealed that live-attenuated *Eimeria *spp. strains contained in the anticoccidial vaccine were effective to replace intestinal replication and spreading of wild *Eimeria *spp. strains. This notion is supported by the fact that vaccinated birds excreted high numbers of oocysts in feces with no effects on animal productivity. Although the beneficial effects of anticoccidial vaccines in poultry are well documented, the high costs associated with this prophylactic approach limit its widespread use in animal production [8]. To this end, the design of novel and not expensive alternatives for the control of coccidiosis is an important task to maintain the productivity of the poultry industry.

The present study revealed that dietary supplementation of 0.05% of YCW increases local mucosal IgA secretions, cellular and humoral immune responses, and reduces parasite excretion in feces of neonatal animals infected with coccidia. These results provide clear evidence of the potential of this dietary strategy as an anticoccidial alternative. Specifically, this study reveals the potential of YCW dietary supplementation to enhance cell mediated immune responses, an important factor for the protection against coccidial infections [4,5,21].

In the present study, dietary supplementation of YCW improved weight gain and feed conversion, and reduces parasite excretion in feces of animals naturally infected with coccidia. Only a few studies have examined the effect of YCW on coccidial infections. Chickens supplemented with yeast-culture residues in the diet and raised on coccidia-infected litters [22]; and chickens fed with YCW and experimentally infected with *Eimeria *spp. [23] showed improved growth performance compared to control treatments. Results of all three reports consistently indicate that dietary supplementation of YCW or derivates could be an important alternative for reducing performance losses provoked by coccidial infection.

The effects of diets supplemented with YCW on systemic and local immune responses have been demonstrated in many animal models including cows [24], pigs [25,26], dogs [27,28], rats [29], chickens [30,31], rainbow trout [32], etc. Likewise, it has been shown that YCW supplemented diets boost immune responses against vaccine antigens; for example, in cows, YCW enhanced humoral and cellular immune responses against rotavirus vaccine [24]. In piglets, YCW improved humoral and cellular immune responses against Aujeszky's disease vaccination [25]. Interestingly, in humans, oral supplementation of a yeast-based product significantly reduced the incidence and duration of influenza outbreaks [33]. In the present study, dietary supplementation of YCW increased the production of antibodies against NDV and IgA concentrations in the intestinal and tracheal mucosa. Moreover, YCW supplementation reduced the number of *Eimeria *spp. oocysts excreted in feces. A similar effect on parasite oocysts excretion was reported elsewhere [34]. Although the specific mechanisms of action by intestinal IgA on coccidial infections are still unknown, it is thought that secretory IgA attaches to the parasite surface and prevents its binding to the intestinal epithelium. Also IgA reduces the development of sporozoites or merozoites and prevents invasion within host cells [35,36]. Interestingly, the present study also revealed that the YCM treatment increased IgA secretions in both the intestinal and tracheal tissues while the vaccine treatment increased this antibody only in the intestinal samples. These observations indicate that YCM treatment stimulate IgA secretions systemically while the vaccine treatment stimulate secretions only in tissues exposed to the parasite. These results, taken together, provide new and clear evidence that YCW has an immune-regulatory effect at the local and systemic level.

It can be hypothesized that the increase in humoral responses induced by YCW supplementation are responsible for the reduction in coccidia replication in the intestinal mucosa [37]. However, recent evidence revealed that humoral immunity plays only a minor role in protection against this disease [4,35]. Cell-mediated immune responses are thought to be the most important factor for protection against coccidiosis. CD4+ and CD8+ T-cells populations limit coccidia replication in the intestinal tract [4,35,38]. The effects of T-cells on *Eimeria *spp. are mediated by the cytokines that these cells release. Interferon-gamma and tumor necrosis factor-alpha limit oocyst production in either primary or secondary infections (for details see reviews [4,35,38]). The immune-regulatory effects of YCW on cell-mediated responses have been confirmed in several human and animal studies [39-42]. Therefore, it is possible to hypothesize that the reduction in *Eimeria *spp. replication in the intestine was caused by the YCW-induction of T-cell responses. Even though T-cells phenotypes were not directly examined in the present study, cutaneous basophilic hypersensitivity test is an excellent and well accepted method for the determination of T-cell response [17,18,43,44]. To our knowledge this study provides the first evidence of the effect of YCW on regulation of T-cell responsiveness, which could be important for immunity against coccidial infections. Further studies will elucidate T-cell repertoire and cytokines responses stimulated by YCW dietary supplementation.

## Conclusion

The present data show that dietary supplementation of YCW in neonatal animals can enhance the immune responses against coccidial infections. Moreover, the present study provides a new dietary strategy for the alternative control of coccidiosis and more importantly, confirms the potential of YCW as adjuvant for modulating mucosal immune responses [45]. Despite the difficulties of designing effective approaches for the control of coccidiosis, this study provides a promising dietary strategy with the potential to enhance the health of immune-compromised subjects and children in developing countries.

## Abbreviations

ANOVA: analysis of variance; ELISA: enzyme-linked immunosorbent assay; IgA: secretory immunoglobulin A; NDV: Newcastle disease virus; NRC: National Research Council; PBS: phosphate-buffered saline solution; PHA-A3: phytohemagglutinin; SEM: standard error of the mean; YCW: yeas cell wall.

## Competing interests

The authors declare that they have no competing interests.

## Authors' contributions

GGV, ACC, CLP, EAG, and GMN designed the study. GGV and ACC performed the experiments. GGV, EAG, and GMN analyzed the data and prepared the manuscript. All authors read and approved the final manuscript.
